# Improvement in Serum Biochemical Alterations and Oxidative Stress of Liver and Pancreas following Use of Royal Jelly in Streptozotocin-Induced Diabetic Rats

**DOI:** 10.22074/cellj.2016.4564

**Published:** 2016-08-24

**Authors:** Elham Ghanbari, Vahid Nejati, Mozafar Khazaei

**Affiliations:** 1 Fertility and Infertility Research Center, Kermanshah University of Medical Sciences, Kermanshah, Iran; 2 Department of Biology, Faculty of Science, Urmia University, Urmia, Iran

**Keywords:** Diabetes Mellitus, Liver, Oxidative Stress, Pancreas, Royal Jelly

## Abstract

**Objective:**

This study aimed to evaluate the effects of royal jelly (RJ) on serum biochemical alterations and oxidative stress status in liver and pancreas of streptozotocin (STZ)- induced diabetic rats.

**Materials and Methods:**

In this experimental study, thirty two male Wistar rats were divided into the following four groups (n=8/group): i. Control (C), ii. Diabetic (D), iii. Royal jelly (R), and iv. Royal jelly-treated diabetic (D/R) groups. Diabetes was induced by single intraperitoneal (IP) injection of STZ (60 mg/kg). The RJ [100 mg/kg body weight (BW)] was administered orally for 42 days. Blood samples were used to determine serum levels of insulin, high density lipoprotein cholesterol (HDL-c), total protein (TP), albumin, alanine aminotransferase (ALT), aspartate aminotransferase (AST), alkaline phosphatase (ALP), and fasting blood glucose (FBG). Also, the antioxidant status was evaluated by determining the levels of malondialdehyde (MDA), catalase (CAT) and ferric reducing antioxidant power (FRAP) in liver and pancreas. Data were analyzed by one-way analysis of variance (ANOVA) with P<0.05 as the significant level.

**Results:**

STZ-induced diabetic rats showed a significant elevation in the serum levels of AST, ALT, ALP and FBG, whereas there was a significant decrease in serum levels of insulin, albumin, HDL-c and TP (P<0.05). Treatment of the diabetic rats with RJ restored the changes of the above parameters to their normal levels (P<0.05). In addition, RJ significantly improved reduced levels of FRAP and CAT as well as high MDA level in liver and pancreas (P<0.05).

**Conclusion:**

RJ improves oxidative damage induced by STZ in the liver and pancreas of rats; therefore, it can be considered as an effective and alternative treatment for diabetes.

## Introduction

High fasting blood glucose (FBG) level is considered as a hallmark feature of diabetes mellitus, owing to a loss or lack of insulin-producing pancreatic β-cells (type Ι diabetes) or via loss of insulin sensitivity in its target tissues like muscle and adipose (type II diabetes) (1). High FBG levels leads to various organs dysfunction, especially eyes, kidneys, livers, heart and blood vessels (2). A significant increase in the FBG level in diabetic rat could be due to β-cells destruction in the pancreatic islets by streptozotocin (STZ) (3). Liver as an insulin-dependent tissue plays a vital role in glucose and lipid homeostasis (4). Oxidative stress causing structural and functional alterations in the cellular biomolecules (such as lipids, nucleic acid, and proteins) and cell membrane is the result of the development of complications in diabetic individuals (5,6). Malondialdehyde (MDA) is an end product of lipid peroxidation that is widely used as an important indicator of cellular injury (7). 

Antioxidant enzymes such as catalase (CAT), superoxide dismutase (SOD) and glutathione peroxidase (GSH-Px) act as free radical scavengers. These antioxidant enzymes as electron donors react with the reactive oxygen species (ROS) to form innocuous products and inactivate the free radicals. Therefore, antioxidants protect cells against oxidative damage (8). These antioxidant enzymes are impaired in diabetes mellitus (9). 

Poor glycemic control may also lead to a decrease in antioxidant defense system that is followed by the damage of cellular organelles and enzymes, increased level of lipid peroxidation, and development of insulin resistance (10,11). 

The liver is severely damaged in patients with diabetes mellitus. These damages include abnormal liver enzymes levels, necrosis, inflammation, cirrhosis, hepatocellular carcinoma, hepatitis, nonalcoholic fatty liver disease (NAFLD) and acute liver failure (12). Fluctuating levels of alkaline phosphatase (ALP), aspartate aminotransferase (AST) and alanine aminotransferase (ALT) are mostly the result of the leakage of these enzymes from the cytosol of hepatocytes into the bloodstream. However, increased levels of these enzymes may result in low levels of serum albumin and total protein (TP) that are used as indicators of liver functions (13). 

Royal jelly (RJ) is composed mainly of protein, fatty acids, vitamins (C and B), sugars and minerals (iron, calcium, copper, potassium, magnesium, zinc and sulfur) (14). It has been shown to possess a variety of pharmacological activities such as hypoglycemic (15), hypotensive (16) and antihypercholesterolemic (17) activities. 

On the other hand, RJ with high antioxidative and scavenging activities counteracts the lipid peroxidation caused by free radicals under *in vitro* condition (18). Previous studies have shown RJ ameliorates insulin resistance in fructose-drinking rats (19). In another study, it has been shown that RJ treatment also increased serum levels of high density lipoprotein (HDL-c) and decreased serum levels of total cholesterol (TC) and triglyceride (TG) in hypercholesterolemic rats (20). There was no scientific report regarding RJ effect on liver and pancreas in STZ-induce diabetic rats; therefore, the aim of present study was to examine the ability of RJ to improve biochemical alterations and oxidative stress status in liver and pancreas of STZinduced diabetic rats. 

## Materials and Methods

### Animals

In this experimental study, thirty two adult male Wistar rats (190 ± 10 g), 10-12 weeks old (Pasteur Institute) were used in Kermanshah University of Medical Sciences, September 2014. Animals were kept according to the Guide for the Use and Care of Laboratory Animals as approved by the Ethics Committee of Kermanshah University of Medical Sciences. Before and during the experiment, the animals were maintained under standard conditions, with a temperature of 22 ± 2˚C, a regular 12/12 hour light-dark cycle. The rats were allowed free access to standard pellet and tap water ad libitum. 

### Experimental induction of diabetes

To induce diabetes mellitus, after 12-hour overnight fasting, the rats received an intraperitoneal (IP) injection of freshly prepared STZ (Sigma, USA) at the dose of 60 mg/kg body weight (BW) in 0.1 mol/L citrate buffer (pH=4.5) (21). On days 3 and 7 after STZ injection, blood samples were collected from tail veins using ACCU-Check Aviva blood glucose meter (Roche, Germany) in order to confirm the diabetes. The rats with FBG levels above 250 mg/dL were only included in diabetic (D) and royal jelly-treated diabetic (D/R) groups. 

### Preparation of the royal jelly

RJ was obtained from a local beekeeping association (Urmia, Iran), and kept at -4˚C until use. The quality of RJ was confirmed by an expert academic member of Urmia University. The RJ was administered at dose of 100 mg/kg BW in distilled water (22) for 6 weeks. Each rat was orally administrated RJ solution at a volume of 1 ml once a day (10:00 to 11:00 AM). The control (C) and diabetic (D) groups received the same volume of distilled water once a day. 

### Experimental and animal design

Male Wistar rats (n=32) were randomly divided into four groups (n=8/group) as follows: i. Control group (C) receiving distilled water (DW) (1 ml/ day) for 6 weeks, ii. Royal jelly group (R) receiving RJ (100 mg/kg) dissolved in 1 ml DW for 6 weeks, iii. Diabetic group (D) receiving DW (1 ml/ day) for 6 weeks, and iv. Royal jelly-treated diabetic group (D/R) receiving RJ for 6 weeks (100 mg/kg/day). 

### Serum biochemical assay

#### Measurement of serum insulin and blood glucose levels

The rats of all groups were anesthetized by an IP injection of ketamine 5% (Razak, Iran) after 12-hour overnight fasting. Blood samples were obtained by cardiac puncture using heparinized syringe (Rotexmedica, Germany). The samples were then centrifuged at 3000 rpm for 15 minutes at 25˚C, while the serum was separated and stored at -70˚C. Serum insulin level was measured using an enzyme-linked immunosorbent assay (ELISA) kit (DRG Instruments, Germany). FBG levels were measured using an ACCU-Check Aviva glucose meter (Roche, Germany) on days 1 and 42 of treatment. 

#### Biochemical parameters determination

The serum levels of AST, ALT, and ALP were determined by a diagnostic kit (DiaSys Diagnostic Systems, Germany). The serum levels of albumin and TP were determined by an automated analyzer (Architect c8000, Clinical Chemistry System, USA) using a reagent kit (Boehringer, Mannheim, Germany) according to the manufacturer’s instructions. 

### Tissue preparation for measurement of oxidative stress parameters

After rats were sacrificed, the liver and pancreas were excised immediately and rinsed with isotonic saline. Liver and pancreas tissues were then minced and a homogenate was prepared with 10% (w/v) phosphate-buffered saline (PBS, 0.1 mol/L, pH=7.4). The homogenate was centrifuged (Sigma, Germany) at 10,000 rpm for 5 minutes at -4˚C. The supernatant was directly used for the determination of MDA, FRAP and CAT levels. 

### Malondialdehyde assay

MDA level was determined spectrophotometrically according to the methods of Rajasekaran and Kalaivani (23). About 0.1 ml of the supernatant was mixed with 2 ml of thiobarbituric acid (TBA)trichloroacetic acid (TCA)-HCl reagent (1:1:1 ratio, 0.37% TBA, 15% TCA and 0.25 N HCl, Sigma, USA). After cooling, a pink color appeared due to MDA-TBA reaction. The absorbance was measured using spectrophotometer (Pharmacia, Novaspec II, Biochrom, England) at 535 nm, and the results were expressed in nmol/mg of protein. 

### Catalase assay

The catalase activities in liver and pancreatic homogenates were measured accordance to the procedure reported by Aebi (24). Briefly, 0.2 ml of tissue homogenate and 1.2 ml of PBS were mixed, but the reaction was started by the addition of 1.0 ml of 30 mM H_2_O_2_ solution. The absorbance was measured using spectrophotometer (Pharmacia, Novaspec II, Biochrom, England) at 240 nm at a 30-second interval. The results were expressed as units per mg tissue. 

### Ferric reducing antioxidant power assay

The ferric reducing antioxidant power (FRAP) assay was used as described previously (25) to assess the ability of the sample to reduce the Fe^3+^-2, 4, 6-tri(2-pyridyl)-s-triazine (TPTZ, Merck, Germany) complex to the ferrous form (Fe^2+^-TPTZ complex) at low pH, forming an intense blue- coloured complex with optimal absorbance at 593 nm. Briefly, TPTZ powder (0.00823 g) was dissolved in 2.5 ml HCl (40 mmol/L) to prepare the TPTZ solution, after which 25 ml of an acetate buffer solution (300 mmol/L, pH=3.6) and 2.5 ml of a FeCl_3_. 6H_2_O solution (20 mmol/L) were mixed with TPTZ solution to prepare fresh FRAP reagent. Then, 1.5 ml of fresh FRAP reagent was mixed with 150 μl tissue homogenates and incubated at 37˚C for 10 minutes. The absorption was measured at 593 nm using a spectrophotometer (Pharmacia, Novaspec II, Biochrom, England). FeSO_4_ was used as a standard of FRAP assay and the results were expressed as µM/mg.

### Statistical analysis 

Data were expressed as mean ± SE. Variations between groups were tested for significance using one way analysis of variance (ANOVA) followed by Tukey’s test. A value of P≤0.05 was regarded as significant. All statistical analyses were performed using the Statistical Package for the Social Sciences (SPSS, SPSS Inc., USA) version 18. 

## Results

### Blood glucose and insulin

**Fig.1:**
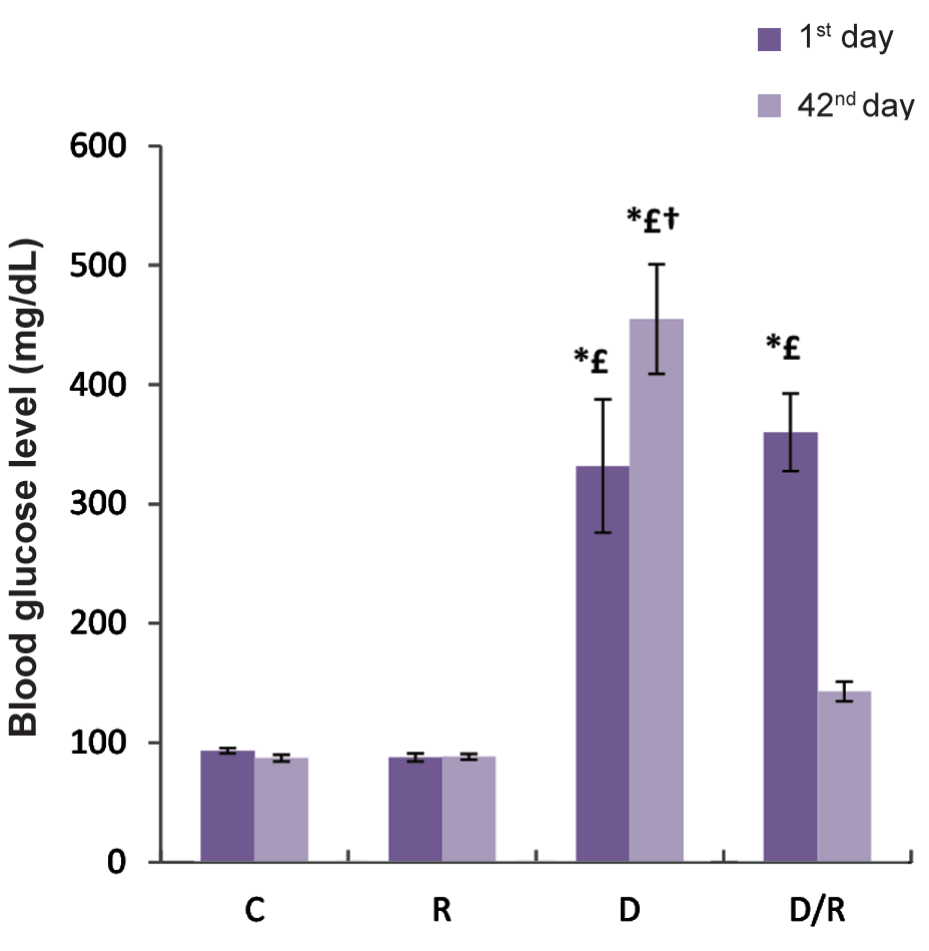
FBG level (mg/dL) of all groups during the treatment period. Results are presented as mean ± SE. *; P<0.05 as compared to the group C, £; P<0.05 as compared to group R, †; P<0.05 as compared to D/R group, FBG; Fasting blood glucose, SE; Standard error, C; Control group, R; Royal jelly group, D; Diabetic group, and D/R; Royal jelly-treated diabetic group.

**Fig.2:**
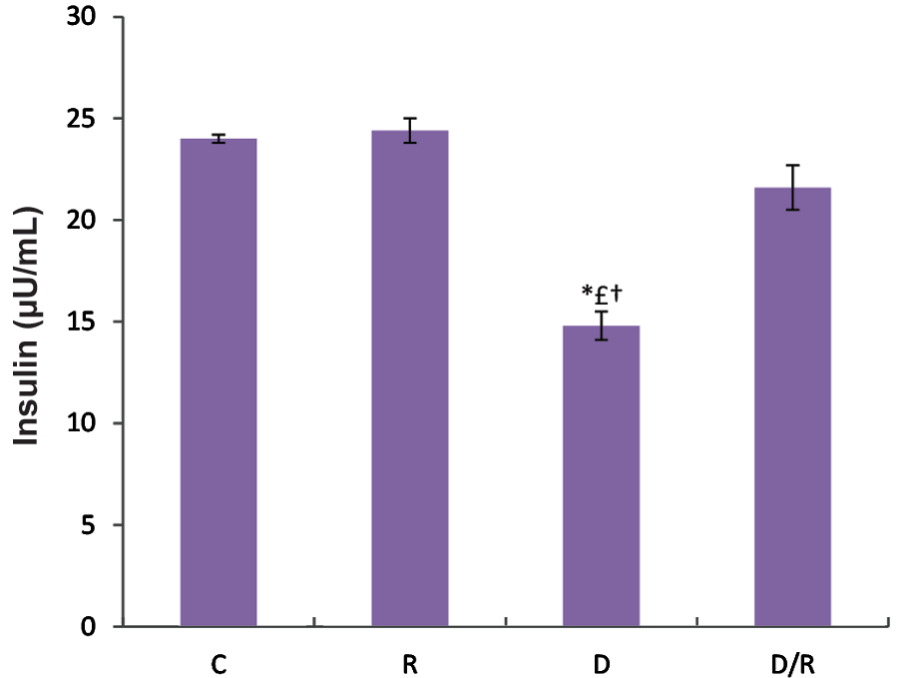
Serum insulin levels in streptozotocin-induced diabetic rats. Values are given as mean ± SE. * £†; P<0.05 as compared to the other groups, SE; Standard error, C; Control group, R; Royal jelly group, D; Diabetic group, and D/R; Royal jelly-treated diabetic group.

There was no significant difference in FBG level among the animals on day 1 of experiment. However, on day 42, there was a significant decrease in the FBG level of the D/R group as compared to the D group (P=0.002, Fig.1). The serum insulin level significantly decreased as a result of diabetes in the D group, which was restored during a 6-week of oral administration of RJ in the D/R group (P=0.000, Fig.2). 

### Biochemical parameters examination

The levels of ALP (P=0.001) and ALT (P=0.000) in the D group were significantly higher than the C group. Serum ALT and ALP levels showed a significant decrease in the D/R group in comparison to the D group, while there was no significant difference regarding the mean concentrations of ALT and ALP in the C and R groups. The serum ALP levels were 95.4 ± 4.88, 85.8 ± 2.21, 170.9 ± 15.18 and 124.4 ± 2.71 ng/dl in the C, R, D and D/R groups, respectively. The serum ALT levels were 24.67 ± 1.83, 23.14 ± 0.89, 57.63 ± 5.69 and 31.67 ± 2.3 ng/dl in the C, R, D and D/R groups, respectively (Table 1). 

AST levels were 94.1 ± 2.6 and 179.7 ± 2.4 U/L in the C and D groups, respectively. This increase owed to oral administration of RJ. ALT level was 114.6 ± 7.04 U/L in the D/R group, indicating a significant difference as compared to the ALT level of D group. Also, a significant difference was determined regarding ALT level between the C and D/R groups (P=0.010, Table 1). 

TP levels were 6.23 ± 0.08, 6.24 ± 0.06, 3.71 ± 0.13 and 5.69 ± 0.13 g/dl in the C, R, D and D/R groups, respectively. Albumin levels were 4.7 ± 0.18, 4.83 ± 0.17, 2.25 ± 0.08 and 3.25 ± 0.09 g/dl in the C, R, D and D/R groups, respectively (Table 2). The results indicated that diabetes caused a significant decrease in serum TP (P=0.000) and albumin levels (P=0.001), but their levels were significantly reversed by the oral administration of RJ. The levels of serum TP and albumin showed no significant differences between the C and R groups. 

In this study, no significant difference was observed in the serum HDL-c levels between the C and R groups. The results demonstrated a significant decrease in the serum HDL-c levels of the D group. A significant elevation in the serum HDL-c level was observed at the end of week 6 of treatment with RJ (100 mg/kg) in the D/R group as compared with the D group (P=0.000, Table 2). HDL-c levels were 0.84 ± 0.015, 0.86 ± 0.01, 0.34 ± 0.018 and 0.68 ± 0.019 mmol/L in the C, R, D and D/R groups, respectively. 

**Table 1:** Comparison of RJ effects on the serum levels of AST, ALP, and ALT among the groups


Groups (n=8/group)	AST (U/L)	ALP (U/L)	ALT (U/L)

C	94.1 ± 2.6	95.4 ± 4.88	24.67 ± 1.83
R	85.4 ± 2.2	85.8 ± 2.21	23.14 ± 0.89
D	179.7 ± 2.4^*£†^	170.9 ± 15.18^*£†^	57.63 ± 5.69^*£†^
D/R	114.6 ± 7.04^*£※^	124.4 ± 2.71	31.67 ± 2.3


Data are expressed as mean ± SE, *; P<0.05 as compared to the group C, £; P<0.05 as compared to the group R, ※; P<0.05 as compared to the group D, †; P<0.05 as compared to D/R Group, ALT; Alanine aminotransferase, AST; Aspartate aminotransferase, ALP; Alkaline phosphatase, SE; Standard error, C; Control group, R; Royal jelly group, D; Diabetic group, and D/R; Royal jelly-treated diabetic group.

**Table 2:** Comparison of the serum levels of HDL, TP, and albumin among the groups C, R, D and D/R


Groups (n=8/group)	HDL (mg/dL)	TP (g/L)	Albumin (g/dL)

C	0.84 ± 0.015	6.23 ± 0.08	4.7 ± 0.18
R	0.86 ± 0.01	6.24 ± 0.06	4.83 ± 0.17
D	0.34 ± 0.018^*£†^	3.71 ± 0.13^*£†^	2.25 ± 0.08^*£†^
D/R	0.68 ± 0.019^*£※^	5.69 ± 0.13^*£※^	3.25 ± 0.09^*£※^


Data are expressed as mean ± SE. *; P<0.05 as compared to the group C, £; P<0.05 as compared to the group R, ※; P<0.05 as compared to the group D, †; P<0.05 as compared to the group D/R, HDL; High den- sity lipoprotein, TP; Total protein, SE; Standard error, C; Control group, R; Royal jelly group, D; Diabetic group, and D/R; Royal jelly-treated diabetic group.

### Malondialdehyde examination

**Fig.3:**
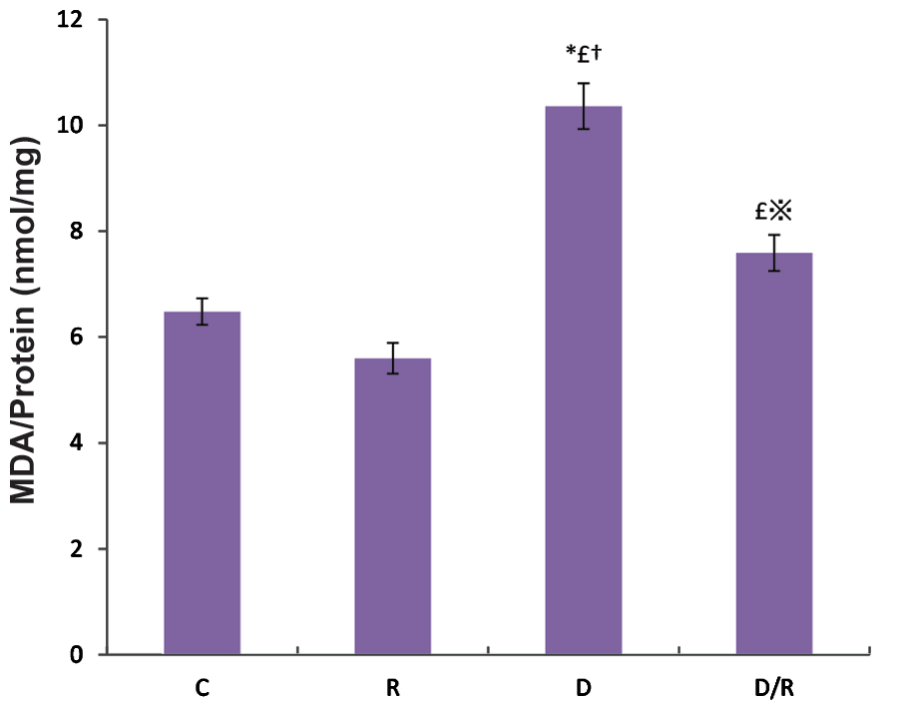
MDA levels in liver of all groups. Values are given as mean ± SE for groups. *; P<0.05 as compared to the group C, £; P<0.05 as compared to the group R, ※; P<0.05 as compared to the group D, †; P<0.05 as compared to the group D/R, MDA; Malondialdehyde, SE; Standard error, C; Control group, R; Royal jelly group, D; Diabetic group, and D/R; Royal jelly-treated diabetic group.

**Fig.4:**
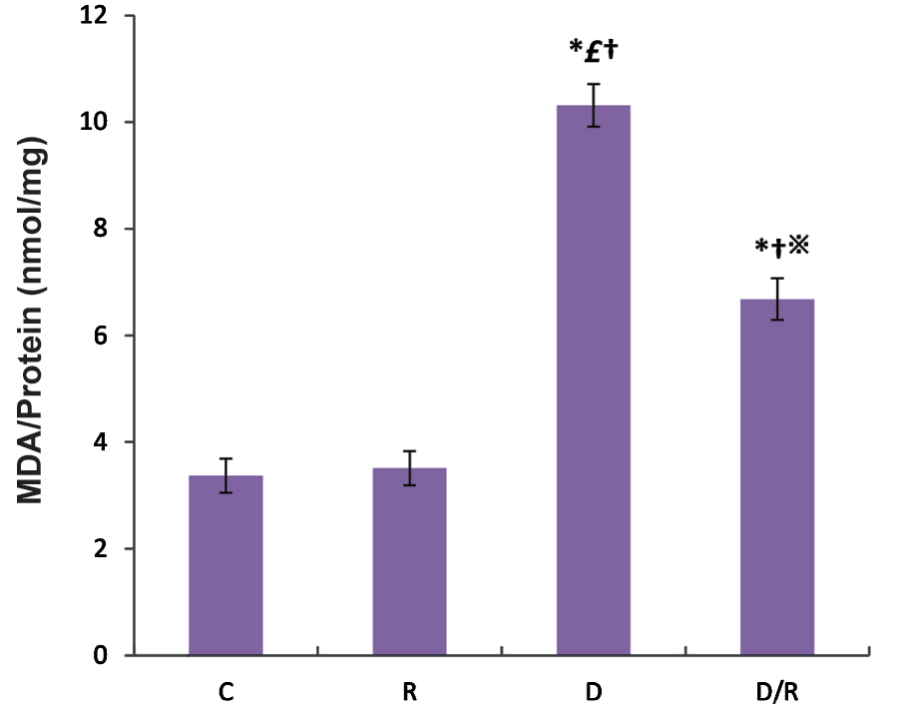
RJ effect (100 mg/kg BW) on pancreas MDA levels of the control and experimental rats. Values are expressed as mean ± SE. *; P<0.05 as compared to the group C, £; P<0.05 as compared to the group R,※; P< 0.05 ascompared to the group D, †; P<0.05 as compared to the D/R group, RJ; Royal jelly, BW; Body weight, MDA; Malondialdehyde, SE; Standard error, C; Control group, R; Royal jelly group, D; Diabetic group, and D/R; Royal jelly-treated diabetic group.

The MDA levels were significantly increased in both the liver (P=0.001, Fig.3) and pancreas (P=0.000, Fig.4) tissues of the D group as compared to the C and R groups. These MDA level in the D/R group was significantly lower than those of the D group. The mean MDA levels of pancreas and liver in the D/R group was significantly higher than the relative value of the R group. 

### Catalase examination

**Fig.5:**
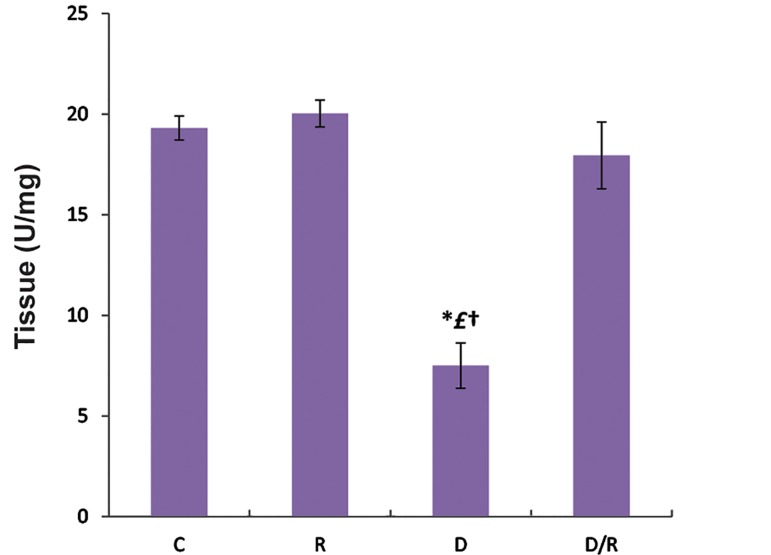
Liver CAT activities of all groups. Data are expressed as mean ± SE. *; P<0.05 as compared to the group C, £; P<0.05 as compared to the group R, †, P<0.05 as compared to the D/R group, CAT; catalase, SE; Standard error, C; Control group, R; Royal jelly group, D; Diabetic group, and D/R; Royal jelly-treated diabetic group.

**Fig.6:**
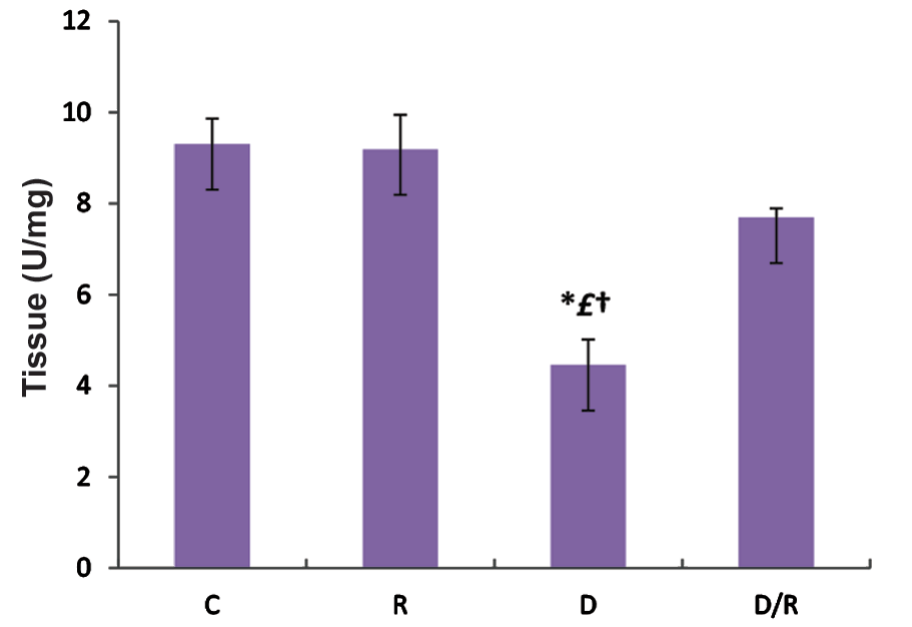
Mean pancreatic CAT activates of all groups. Data are expressed as mean ± SE. *; P<0.05 as compared to the group C, £; P<0.05 as compared to the group R, †, P<0.05 as compared to the D/R group, CAT; Catalase, SE; Standard error, C; Control group, R; Royal jelly group, D; Diabetic group, and D/R; Royal jelly-treated diabetic group.

The mean CAT activities of liver (P=0.000, Fig.5) and pancreas (P=0.001, Fig.6) of the D/R group were significantly higher than D group. CAT activities of liver and pancreas of the D group were lower than those of the C and R groups. There was no significant change in CAT activities of liver and pancreas tissues among C, R and D/R groups. 

### Ferric reduce antioxidant power examination 

**Fig.7:**
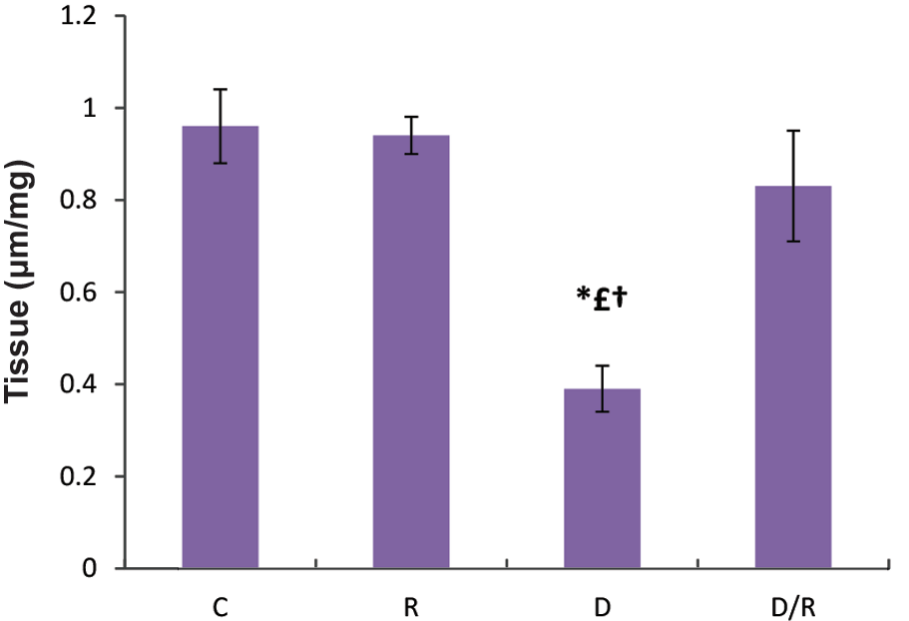
FRAP level in liver of all groups. Data are expressed as mean ± SE. *; P<0.05 as compared to the group C, £; P<0.05 as compared to the group R, †; P<0.05 as compared to the D/R group, FRAP; Ferric reducing antioxidant power, SE; Standard error, C; Control group, R; Royal jelly group, D; Diabetic group, and D/R; Royal jelly-treated diabetic group.

**Fig.8:**
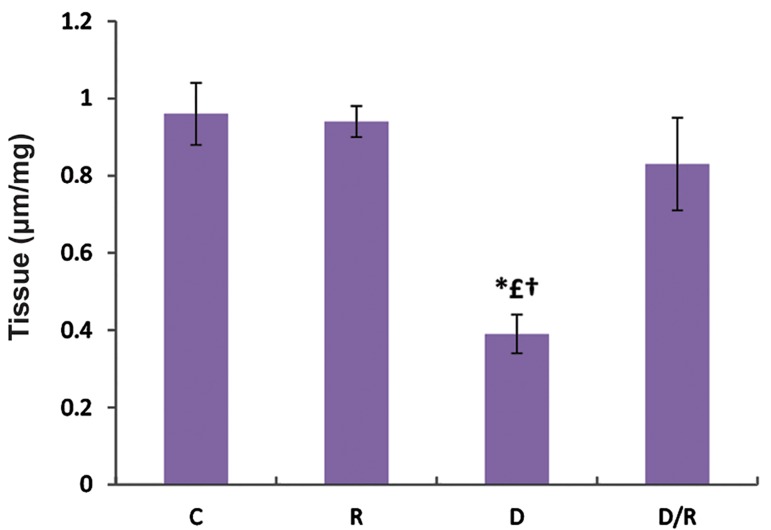
FRAP level in pancreas of all groups. Data were given as mean ± SE. One way ANOVA is followed by Tukey’s test. *; P<0.05 as compared to the group C, £; P<0.05 as compared to the group R, †; P<0.05 as compared to the D/R group, FRAP; Ferric reducing antioxidant power, SE; Standard error, C; Control group, R; Royal jelly group, D; Diabetic group, and D/R; Royal jelly-treated diabetic group.

The FRAPs levels of liver (P=0.000, Fig.7) and pancreas (P=0.010, Fig.8) showed a significant decrease in the D group. There was a significant increase in FRAP levels of the D/R group as compared to the D group. There was no statistically significant difference regarding FRAP levels among the C, R and D/R groups. 

## Discussion

We demonstrated that oral administration of RJ improves serum biochemical levels of AST, ALT, ALP, TP, HDL-c, FBG, albumin and insulin, as well as oxidative stress status in pancreas and liver of STZ-induced diabetic rats. To our knowledge, the current study is the first report regarding the effectiveness of RJ on the biomarkers levels and antioxidant status of liver and pancreas in STZinduced diabetic rats. 

A significant increase in the FBG level in STZ-induced diabetic rats, demonstrated in present study, was the same as the previous studies (26,27). Furthermore, another study has reported that fasting hyperglycemia caused a significant decrease in the serum insulin levels due to the damage caused by STZ of β cells in the islets of Langerhans (28). Similarly, our findings revealed that diabetes led to remarkable decrease in serum insulin levels. Zamami et al. (19) have reported that RJ may improve insulin resistance via antioxidant properties. Thus, it may be effective to chronic hyperglycemia associated with diabetes. 

Münstedt et al. (15) have reported that RJ decreases FBG levels in healthy subjects and also believed that insulin like peptides of RJ can protect its activity even after passage through human stomach. Also, in present studies, RJ treatment caused a significant decrease in the elevated FBG level and a significant increase in serum insulin concentration after 6 weeks of treatment in diabetic rats. These findings revealed the ability of RJ for modulating disorders caused by diabetes. 

A decrease in serum albumin and TP levels may contribute to the inhibition of oxidative phosphorylation process, leading to a reduction in protein absorption, a decline in protein synthesis, and an increase in the catabolic process (29). In the present study, a significant decrease in serum albumin and TP levels and an increase in the markers of liver injury (ALP, AST and ALT) reflected the hepatocytes damage in STZ-induced diabetic rats. This finding is consistent with the results indicated by Ramesh et al. (30) and El-Demerdash et al. (31). On the other hand, we found that RJ improved serum biomarkers (ALT, AST and ALP) levels of liver damage. Also, albumin and TP levels were increased in diabetic rats treated by RJ, indicating the hepatoprotective properties of RJ. 

This study supported the findings of previous reports which have indicated the co-treatment of cisplatin and RJ resulted in a significant trend to normal values of ALT, AST, TC, TG, total bilirubin and TP levels as compared to the control rats (32). Wang et al. (33) have showed that the serum HDL-c level is low in STZ-induced diabetic rats. We demonstrated RJ treatment was effective in preventing diabetes causing a decrease in serum HDL-c level. Our findings are in accordance with Cakatay and Kayali (34) as they have showed diabetes can significantly decrease plasma FRAP levels. Also, Ghanbari et al. (35) have reported that oxidative induction by diabetes decreases the FRAP level in kidney tissues, whereas after treatment with RJ, the FRAP levels were significantly increased. The present study implied that treatment with RJ significantly enhanced the FRAP levels in liver and pancreas tissues of diabetic rats. 

Kanbur et al. (36) have suggested that RJ treatment reduced MDA levels in liver of paracetamolinduced liver damage rats. Also, El-Nekeety et al. (37) have reported that RJ reduced MDA levels in fumonisin rat. We also demonstrated that RJ treatment of diabetic rats significantly decreased the MDA levels in liver and pancreas tissues. 

The present findings showed a significant decrease in CAT activity in pancreatic tissue of diabetic rats. These finding is supported by the result of Ramachandran et al. (38). Cihan et al. (39) have indicated that RJ caused a significant elevation in the activities of GSH-Px, SOD and CAT of hepatic tissue in radiationinduced oxidative stress in rats. In the present study, a decrease in the CAT activity suggested that oxidative stress was increased in diabetic rat, and a significant elevation of CAT activity and FRAP levels were observed in the RJ-treated diabetic rats which is likely due to its free-radical-scavenging activity. 

## Conclusion

Protective effect of RJ against diabetes is due to a reduction in MDA level and improvement of antioxidants status in liver and pancreas tissues. Oral administration of RJ improves the serum levels of AST, ALT, ALP, TP, HDL-c, insulin, FBG and albumin in STZ-induced diabetes. 
